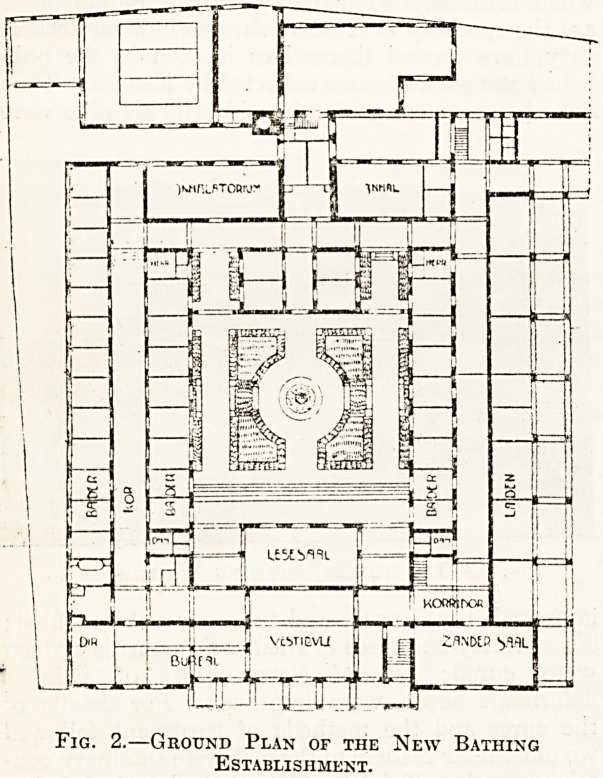# Home and Foreign Spas

**Published:** 1912-08-10

**Authors:** 


### HOME AND FOREIGN SPAS.*

### XI.—Bad-Kreuznach.

An old-fashioned, picturesque town is Kreuznach, lying on both banks of the river Nahe, some distance before it flows into the wider stream of the Rhine. The special part of the town which is devoted to the bathing establishment—Bad-Kreuznach—is situated partly on an island and partly on the right bank of the Nahe, and during recent years the municipality has done its utmost to make it a thoroughly up-to-date and satisfactory spa. As such it has undoubtedly attractions which should win it a well-deserved popularity among English visitors, who will find it a quiet, delightful place in which to pass a couple of weeks. While it is devoid of the fashionable activity which is such a feature of the larger Continental spas, it may claim to offer the patient who decides to try it special attractions of its own which do more than compensate for the want and absence of luxury. Around it is to be found the most splendid scenery that the Rhine valley offers; the patient who can take walks and excursions will find a number of interesting sites which will well repay attention; the camera-lover will meet with innumerable bits that are fully worth the immortality of the photographic album; the nature-lover, again, will discover that the neighbourhood teems with riches.

### The Problem of Climate.

From a patient's point of view, the most important point in deciding upon the claims of relatively equally endowed spas is the question of climate. Kreuznach delights in the possession of a mild agreeable climate, which in summer is apt to prove too hot for the average English visitor; in autumn and spring, however, the town is perhaps at its best, and anyone who has experienced the pleasures and conveniences which it then affords will readily agree that the spa is one of the most interesting which can be visited on the Continent.

Kreuznach waters possess distinctly radio-active properties, which have been carefully investigated, so that to-day the indications for this particular spring are well understood. The main spring contains about 10 per mille of sodium chloride, and about 2 per mille of calcium chloride, with which are combined minute quantities of other chlorides, while in addition analysis reveals traces of iodides and bromides of the alkaline earth-metals and of arsenic. The cold springs, the chief of which is the Elizabethquelle, are used both for drinking and for bathing, patients taking several glasses of the water on an empty stomach early in the morning. The taste of the water is by no means unpleasant, though the saltish taste is at first very pronounced, especially in the tepid waters.

The new central baths are situated close to the Kurhaus and park, and cover an area of 2,400 square metres. They are excellently fitted up, and one of their special features is a fine Zander institute, which is provided with the latest apparatus for passive and active movements. The attached ground plan gives some idea of the general arrangement of the baths.

### The Radium Baths.

There are large and spacious reading and resting rooms, with terraces which overlook the ornamental courtyard. The bathing cells are admirably equipped, and range from the state-rooms, known as the Furstenbader, with comfortable salons attached to the small cabinets in which mud-baths and ordinary hip-baths are given. Basins for special radium-baths have been sunk in the floor, so as to avoid any loss of gas in letting in the brine which contains the emanation. These radium baths are the specialty at Kreuznach, and how serviceable they have proved themselves is seen in the published statistics of cases collected by Kemen. Thus out of twenty cases of uric acid arthritis nine were

Fig. 1.—The valley, showing Waterworks.

improved, nine cured, and two only not benefited; out of thirty-one cases of rheumatic arthritis sixteen were cured; out of eleven cases of arthritis deformans seven were improved. For details of the cures and the methods of treatment followed we must refer readers to the elaborate summary contained in the official booklet, "Radiological Information," published by the Kreuznach Medical Society, and obtainable on application to the London Offices of the Spa at the Old Jewry. This interesting booklet contains original articles by Drs. Eichholz, Mesernitzky, Markwald, Kemen, Engelmann, and Director Neumann, and gives full details of the manner in which the radio-active properties of the Kreuznach waters are utilised in the treatment of chronic and subacute affections of the joints.

### The Scrofula and Rickets Treatment.

It is not only in arthritic cases, however, that the waters of Kreuznach are valuable. According to Sir Herman Weber, scrofula and rickets are the

* Previous articles of this series appeared in The Hospital of Jan. 23, Feb. 25, March 25, April 22, May 20, June 3, June 17, July 8, July 29, and August 19, 1911.

chief disorders treated with marked benefit at this spa. Kreuznach possesses a model children's hospital, the Victoria Krankenhaus, where many hundreds of children are annually treated with excellent results. Catarrhal conditions of the respiratory passages are also likely to be bettered by a course of treatment at Kreuznach; the admirable arrangements made for the treatment of such chronic cases as bronchiectasis and chronic bronchitis are an example to many other spas where such cases receive scant consideration. Chronic catarrhal and chronic inflammatory conditions of the female generative organs are also treated here with marked success, and the results recently obtained by employing the radio-active waters as adjuvants to the older methods are such as to justify the hope of a cure in most cases which have not proved amenable to other

Fig. 2.—Ground Plan of the New Bathing Establishment.

methods. Chronic arthritic conditions are also treated with marked success.

Officially the following are among the "indications" given for this spa: Chronic inflammation of the bone, joints, and intestines; scrofula, tuberculous and rheumatic processes; neuralgia, ischia, inflammation of the womb, ovaries, ligaments, and peritoneum; chronic suppuration of the nose and ear; chronic inflammation of the eye, cornea, and lids, *i.e.* of a scrofulous nature; similar chronic catarrh of the respiratory organs, the bladder, urinary passage, womb, and vagina; skin diseases; weakness from age, exhaustion after illness and operations; bacterial diseases; swelling of muscular tissue, new formation of a mild as well as of a malignant nature, fibroid tumour, nyom of the womb and small skin-swellings.

The Kreuznach season commences in May and ends in September, but during the winter months baths and treatment may be obtained, and many English patients will find it preferable to visit the spa in the colder months when the heat is not so oppressive. In that case special arrangements must be made for treatment, but the list of medical men who are willing to undertake the charge of patients at their homes is a long one, and no difficulty will be found in this respect. There are several excellent hotels wherein the standard of comfort is singularly high in proportion to the moderate rates charged. A large number of private boarding-houses and pensions cater for the visitors who desire to stay for a prolonged period and to live as cheaply as possible, while the list of lodgings and private apartments available for the accommodation of visitors is an extensive one. During the season the visitor will find that the management of the baths has made every effort to provide him with legitimate recreations and amusements. The Kurhaus and the fine Kurpark are the chief centres of attraction; bands, concerts, entertainments, games and sports are arranged for in regular succession. There is a fine orchestra, and excellent provision has been made for the children in the shape of large playing grounds, while the more active patients will find a series of tennis courts, and even a racing track available for them. The Kur charges and general rates are comparatively low, and special rebates are granted to members of the medical profession and their families.

### The Best Route.

The spa is reached in fifteen hours from London by the Ostend-Dover route, in seventeen by the Flushing, and in twenty by the Dover-Calais route. Invalids will probably find the Queenborough-Flushing route the most comfortable, and it is also the cheapest. An illustrated pamphlet, giving full details of the baths, of the various methods of treatment employed, of the charges made, and of the attractions, together with a list of the practising physicians, is issued by the Kur Administration, and may be obtained free on application to the London Agency at 24 Old Jewry.

## Figures and Tables

**Fig. 1. f1:**
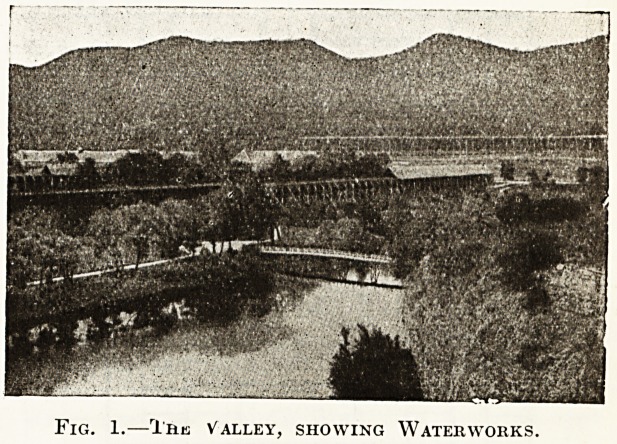


**Fig. 2. f2:**